# #Climatechange vs. #Globalwarming: Characterizing Two Competing Climate Discourses on Twitter with Semantic Network and Temporal Analyses

**DOI:** 10.3390/ijerph17031062

**Published:** 2020-02-07

**Authors:** Wen Shi, Haohuan Fu, Peinan Wang, Changfeng Chen, Jie Xiong

**Affiliations:** 1 Ministry of Education Key Laboratory for Earth System Modeling, Department of Earth System Science, Tsinghua University, Beijing 100084, China; shi-w18@mails.tsinghua.edu.cn (W.S.); haohuan@tsinghua.edu.cn (H.F.); 2 National Supercomputing Center in Wuxi, Wuxi 214000, China; 3 School of Journalism and Communication, Tsinghua University, Beijing 100084, China; wpn17@mails.tsinghua.edu.cn (P.W.); chencf@mail.tsinghua.edu.cn (C.C.); 4 Strategy and Innovation Department, Rennes School of Business, 35065 Rennes, France

**Keywords:** climate change, global warming, semantic network analysis, temporal analysis, public discourse, Twitter

## Abstract

Distinct perceptions of the global climate is one of the factors preventing society from achieving consensus or taking collaborative actions on this issue. The public has not even reached an agreement on the naming of the global concern, showing preference for either “climate change” or “global warming”, and few previous studies have addressed these two competing discourses resulting from distinct climate concerns by differently linking numerous climate concepts. Based on the 6,662,478 tweets containing #climatechange or #globalwarming generated between 1 January 2009 and 31 December 2018, we constructed the semantic networks of the two discourses and examined their evolution over the decade. The findings indicate that climate change demonstrated a more scientific perspective and showed an attempt to condense climate discussions rather than diffuse the topic by frequently addressing sub-topics simultaneously. Global warming triggered more political responses and showed a greater connection with phenomena. Temporal analysis suggests that traditional political discussions were gradually fading in both discourses but more recently started to revive in the form of discourse alliance in the climate change discourse. The associations between global warming and weather abnormalitiessuddenly strengthened around 2012. Climate change is becoming more dominant than global warming in public discussions. Although two discourses have shown more similarities in the rank order of important climate concepts, apparent disagreements continue about how these concepts are associated. These findings lay the groundwork for researchers and communicators to narrow the discrepancy between diverse climate perceptions.

## 1. Introduction

The public’s distinct understanding of the cause and effect of the global climate issue is an obstacle to joint mitigation actions. In addition to a diversity of views co-existing in the public discourse [1,2], previous studies noticed that the public had even failed to reach an agreement on whether “climate change” or “global warming” is the most appropriate definition of the global climate concern [3,4,5]. According to the definition provided by [6], global warming describes global climate issues as a continuous increase in the average temperature of Earth’s surface due to anthropogenic emissions of greenhouse gases, whereas climate change includes not only temperature rise but also a range of complex changes in the state of the climate [7], which may be caused by natural process, external forces, or human interventions [8]. By randomly assigning respondents to climate change or global warming questionnaires, scholars confirmed that the different connotations contained in the two definitions are likely to evoke distinct interpretations of the causes and impacts of the global climate issue [9], which may inhibit collaboration and joint efforts to mitigate the global challenge.

Public preference between climate change and global warming is even more apparent when considering the ideology spectrum [10]. Some scholars concluded that conservatives, who are less concerned with environmental issues, tended to use global warming as a narrative strategy because global warming has a more direct connection with temperature rise, making it easier to find contradictory cues such as freezing weather or heavy snowstorms to deny global climate change facts [11]. The associations between global warming and human activities may contribute to more controversies as well [12], connecting global warming more with the “hoax” frame [5] and evoking greater negative sentiment [13].

Although these existing studies have often attempted to identify the differences between these two terminologies, only a particular few perspectives, such as sentiment, ideological preference, or cause and effect, were examined in each study [3,9,13]. However, the associate network model introduced by psychologists suggests that human recognition and memory have a network-shaped architecture [14], where individual understanding of particular objects is connected with numerous other objects in the mind. According to the associate network model, individual understanding of the global climate concern is a network composed of numerous inter-connected concepts, in which climate change and global warming. As the two terminologies concern the primary mechanism of the global climate issue, the preference between the two understandings may represent two distinct climate discourses by differently organizing numerous climate concepts. Examining the differences between two discourses with an associative perspective may provide communicators with unique insights into narrowing the cognitive discrepancy. The temporal dimension was lacking in existing studies, necessitating the study of how concepts associated with each other have evolved with time.

Large amounts of user-generated data on social media, which have been valued in computer science, communication, and environmental studies [5,9,15,16,17,18], have enabled the acquistion of the social media representation of the two discourses in a decade. In this study, by analyzing hashtag co-occurrence patterns in 6,662,478 tweets containing “climate change” and “global warming” between 1 January 2009 and 31 December 2018, two semantic networks of public climate discourse were constructed to identify the critical concepts and links surrounding the two terminologies. We conducted temporal analysis to observe the evolution of the two discourses and to measure whether the discrepancy between the two has widened or narrowed within the 10-year period.

To be specific, we formulated three research questions (RQs) to be explored in this study:

RQ1: What is the difference in how the two the discourses are associated with important climate concepts in people’s minds?
RQ2: How did the two competing climate discourses evolve from 2009 to 2018?
RQ3: Did the two competing discourses converge or diverge in this decade?

## 2. Background

### 2.1. Climate Change, Global Warming, and Frames

Existing studies have noted that the subtle difference between climate change and global warming evokes different public cognitive responses, where global warming“indicates heat-related impacts, human causes, increased UV light penetration, ozone depletion, and the greenhouse effect, whereas climate change is more associated with a wide range of influences on climate, including drought and agriculture [9]. An N-gram analysis suggested that global warming showed a closer connection with ice, snow, and sea, whereas climate change was always connected with scientific investigations, such as reports, the environment, and science [13]. Some respondents even hold the belief that global warming results in climate change [9].

The two distinct climate discourses being produced based on the same reality can be explained by the framing theory in communication study. Framing refers to the phenomenon where the reality is always partially selected or highlighted when described by the public or media [19]. By distinctly defining problems, suggesting solutions, and indicating casual interpretations [20], different frames tell the audience different stories and influence how they observe facts [21,22]. Two types of frames, equivalency frames and emphasis frames, are commonly studied by scholars to examine how framing effects influence individuals’ attitudes and beliefs [23]. Equivalency frames describe the same fact or logic with different words and may suggest that the audience perceives facts in psychologicallydifferent ways [24]. For example, a cup can be described as “half full” and “half empty”, where the former is a positive frame indicating a reference point lower than current status, and the latter is negative, meaning that the reference point is above the current situation [25]. Emphasis frames employ words selectively associated with parts of reality to shift the audience’s attention to particular attributes [26]. Climate change and global warming have been noted to highlight different aspects of an issue by activating distinct cognitive accessibility patterns [27].

Different frames concerning the global climate concern are popular among the public, politicians, environmentalists, and the media [1,28,29]. Big data analyses have indicated that when interpreting climate events, individuals’ preference for frameworks was influenced by demographics [5] and social-political background [2]. Different choices of frameworks can evoke different psychological processes [30], promote or inhibit engagement intentions [31], or gain approval on various levels [32].

Studies have noted that the frameworks of climate change and global warming may result from different political indications. The American Republican-leaning states show more preference for global warming than climate change compared with Democratic-leaning states, and global warming is more connected with “hoax” in questioning the reality of the global climate issue [5]. Conservatives are more likely to link heat-related phenomena to global warming, whereas liberals associate these facts equally with both frames [27]. An earlier survey conducted by [4] argued that wording choice might not influence the whole population similarly. For the whole sample and politically independent individuals, the two terminologies were equally serious, but climate change seemed more serious compared with global warming among the Republicans, and the Democrats held the opposite opinion.

### 2.2. Network Model for Cognition

Different framework choices may create even more differences than have already been noticed. Psychologists think that human beings are a collection of learned associations [33], and associative response rather than simply linear logic form the structural basis of thought [34]. Associative learning [35] is a long-standing assumption underlying cognitive science [14], suggesting that human cognition toward the world forms a network pattern, where the world is organized into several groups of related items and stored in a network model in the mind. When messages are processed by humans, they are first encoded into a temporary memory network and then linked to an existing associative memory network for long-term storage [36]. In the network, a node represents a certain concept, and edges refers to particular relationships, such as time sequences [37], similarity [38], semantic connections [37], or cause and effect [33] between two nodes.

When individuals search their memory for a particular piece of a message in their mind, the targeted node becomes salient and activated in the temporary memory [39]. If two messages are always activated simultaneously, their connection tends to be more robust and the messages are regarded as associated [36]. If a link is recorded between two concepts, activations are likely to spread through the link from one concept to another with or without conscious awareness [40]. Whereas associations of nodes in the mind may not necessarily reflect the actual relationships of objects, in reality, several factors, including media usage, personal experience, and political stance [34,41,42], may help bundle different sets of concepts.

The hypothesis proposed by [43] suggests that language production may reflect lexical association, and the more often two concepts are simultaneously mentioned in a piece of text, the higher the chance that the two concepts are associated in the individual’s cognitive map of the world. Therefore, semantic network analysis, a methodology based on cognitive science, could help researchers infer the hidden cognitive network structure in the human mind [44] based on the analysis of texts, as the frequency, semantic distance, and co-occurrence pattern of words can accurately reflect their meaning and links [45]. Several researchers have attempted to build a semantic network of certain climate events in social media discourse and scientific publications [46,47,48]. Research about public perceptions of global climate change in Qatar confirmed that the observed semantic network of climate concepts is not random and represents as meaningful clusters [49].

### 2.3. Hashtags as Frame Vehicles on Social Media

Large amounts of unprompted user-generated data on social media serve as distributed sensors that better reflect public opinion [50] and offer researchers more accurate insight into the associations of concepts in people’s minds [51]. In particular, hashtags, a unique mechanism of social media, provide researchers with an unprecedented chance to accurately extract the public cognitive framework contained in texts. To inform or comment [52], social media users attach user-generated tagging with a hash character “#” to anchor the keywords of their tweet and classify the tweet to a unifying topic, and other users can view hashtags as beacons to follow specific topics and join collective discussions. As a communicative marker, hashtags have been used in a wide range of cases [53] and have contributed considerably to conversations and social participation [54,55]. In addition to the function as a topical signifier [56], social scientists noted that hashtags can also represent the context of a tweet [57], flag an individual’s community membership [58], or indicate shared interests [59]. Several studies adopted hashtags as frame markers for both content and sentiment analysis [60,61,62,63,64,65] to eliminate researchers’ subjectivity in frame detection.

In some cases, social media users have long struggled to define the most appropriate hashtag for certain events [60,66]. By observing the usage pattern of trending hashtags “#CrimingWhileWhite” and “#AliveWhileBlack” in a struggle against racism, [67] discovered that two related hashtags generated in a single event will draw public attention to different sides of a story and cause noticeable structural and linguistic differences in public discourse.

When hashtags are used in combination with each other, their co-occurrence pattern in big data can hint to the cognitive associations in people’s thoughts [60,68]. Researchers can extract nonlinear social media narratives [69] and discover the networked frame crowdsourced by the public [61]. In the field of climate communication, co-occurrence of environment and social-political hashtags is thought to strengthen the associations between a variety of trans-regional issues in protests and build collective action frames [70]. The role of climate change issues in the Australian federal election was also discovered with a co-hash analysis [71]. Reference [72] examined how hashtags concerning science, political campaigns, geographical discussions, societal concerns, and new technologies co-occurred with “IPCC”, offering a broad context for studying IPCC communication on social media.

There are many other words in tweets besides hashtags to express the author’s intention. Multiple approaches, such as LDA and STM [32,73], can help to extract topics from unstructured texts. But in this study, targeting on hashtags is more in line with our research question. Firstly, hashtags were invented spontaneously by users of Twitter in 2007 as a mechanism to categorize discussions [74]. Words with hashtags are recognized as topics and considered worthy of public discussion. Secondly, by attaching # to certain words in tweets, the users intentionally anchor their tweets to certain topics. The operator # explicitly reflects the author’s emphasis, which can help us extract rather than infer the author’s identification of the topic of the tweets. Our research question is to analyze and visualize the associations of topics in public climate discourse. Compared with other approaches, analyzing hashtags co-occurrence pattern has advantage in extracting the structure of public discussions.

## 3. Methods

### 3.1. Data Source

As Twitter has been recognized as a popular discussion forum [75] and a social activity platform [76] for climate issues, we followed the literature [5,8,18] and used tweets to investigate distinct perceptions of climate issues and evolution on social media. Although Twitter’s ecosystem has been changing in terms of the number of active users, user demographics, and tweeting conventions in the past years [77,78], the problem is unavoidable for all the information ecosystems on the Internet. As Twitter is one of the most popular social websites, we defined our study as characterizing the perception of climate issues among social media users rather than all the netizens or the whole population.

### 3.2. Data

In this research, we were interested in tweets containing either #climatechange or #globalwarming, as these two hashtags exactly correspond to climate change and global warming, respectively, the two competing definitions of climate issues. We did not follow [79] to include #AGW (anthropogenic global warming) as query hashtags in our research because we think that this refers to global warming in a defined category so cannot be regarded in parallel with the two considered hashtags. We limited the scope of the search to English-language tweets generated between 1 January 2009 and 31 December 2018. We only collected tweets containing either of the two hashtags in the body of the tweets rather than those containing these hashtags in the retweeted or quoted text, as we think that retweeted text or quoted texts cannot directly represent the tweeter’s usage pattern of the two terminologies.

To collect these tweets, we used a Python-based crawler to send requests to the Twitter server to select hashtags, language, start date, and end date as inputs. Once the first request was completed, the server responded with a file in json format and the first 20 qualified tweets in a time-descending order. By parsing the json file, we obtained a string for the crawler to build the next request and obtain the next 20 tweets. Thus, a loop was written to keep the crawler sending requests and the crawler was automatically terminated when all the qualified tweets publicly available were collected. Our crawler respected Twitter’s robot.txt and we did not collect, analyze or display any user information in our study.

**Figure 1:**
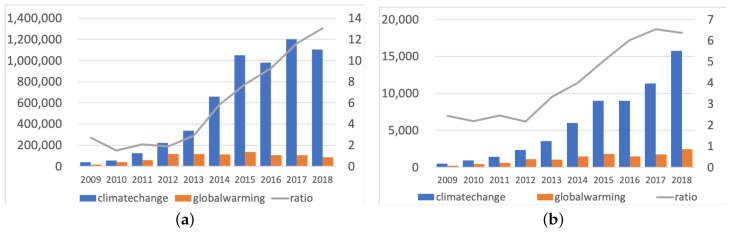
The number of tweets containing #climatechange or #globalwarming, and their ratio from 2009 to 2018 (**a**). The number of hashtags contained in the “climate change” or “global warming” datasets, and their ratio from 2009 to 2018 (**b**).

Given our goal of exploring the difference between the two discourses, the 615,816 tweets containing both hashtags simultaneously were excluded to differentiate between the two datasets following [67,80]. A total of 6,662,478 tweets were retained, of which 5,774,747 contained #climatechange, and 887,731 contained “#globalwarming”. The number of qualified tweets containing #climatechange and #globalwarming in each year is displayed in Figure 1a.

All the hashtags in the tweets were automatically extracted with the Regular Expression Library in Python. Hashtags were transformed to lowercase letters, and clear synonyms were stemmed (e.g., #trump, #DonaldTrump, #donaldtrump). As all the tweets in the “climate change” dataset contained the #climatechange hashtag and all the tweets in the “global warming” dataset contained the #globalwarming hashtag, we did not document these two hashtags when processing data. The number of hashtags contained in the two discourses in each year is displayed in Figure 1b. Hashtags whose frequency was lower than ten times are excluded in the network analysis. As hashtags are intended to be a topic anchor [52], extremely low frequency means that the hashtag is not recognized socially, and excluding them helps researchers focus on meaningful rather than occasional associations.

### 3.3. Measurement

#### 3.3.1. Hashtag Co-Occurrence Network

The co-occurrence patterns of hashtags in tweets from two datasets were documented to build semantic networks for climate change and global warming. For instance, for “#cimatechange redistributes #fish species at high latitudes. @_OScience @AarhusUni #Arctic”, a tweet in the climate change dataset, hashtags #fish and #arctic were documented as co-occurring and their associations plus one in the semantic network of climate change. In the semantic network, nodes represent hashtags and the weight of edge refers to the frequency at which two hashtags co-occurred.

We visualized the network using Gephi software [81]. Following the established literature [60,61,82], only the most prominent hashtags were included in the visualization to concentrate our analysis on the most important hashtags. In this research, the top 50 hashtags with the highest centrality in each network were selected for visualization. Modularity analysis was then analyzed to identify the clusters of hashtags in each semantic network, and hashtags belonging to the same cluster were drawn in the same color. The network spatialization was conducted with Gephi’s built-in force-directed layout algorithm proposed by Fruchterman and Reingold [83], where the more associated the hashtags, the closer they are to each other in the spatial layout.

#### 3.3.2. Temporal Analysis

A temporal analysis was introduced to understand the evolution of the two climate discourses over a long period. We first examined how the two semantic networks evolved in the past years. All the nodes once ranked top 50 in any of the 10 years were gathered to form a union set for each dataset. Then, they were clustered according to the strength of their associations in the whole dataset and mapped with a force-directed layout algorithm in Gephi to produce a graph of nodes. With the dynamic network function supplied by Gephi, we then added the associations between the nodes ranked on the top 50 list in 2009 to the graph of nodes and obtained the relationship of the top 50 nodes for 2009. Similarly, we produced a total of 10 graphs from 2009 to 2018, where the positions of the nodes on the 10 maps are the same, but the strengths of their associations are different to represent the changes in the associations of key hashtags for each discourse.

The correlation between climate change and global warming discourses was measured every year to observe whether the two discourses converged or diverged over time. Considering computing power limitations, only key hashtags ranked in either of the top 50 lists for the two discourses in that year were included in the calculations. First, we measured to what extent the two discourses resemble each other in the order of importance for the hashtags in each year. For every year, the top 50 hashtags in each network were selected with a rank order according to their centrality. Then, Spearman’s rank correlation coefficient was used to examine the correlation of the rank orders of the selected nodes in the two discourses [84], where a high Spearman correlation indicates that the hashtags in the two discourses were ranked similarly. Secondly, we measured to what extent the two discourses resembled each other in the associations between the key hashtags for each year. For every year, we obtained the union of the two top 50 nodes lists and used the name of the nodes in the union as the row name and column name to create two matrices. One matrix was created for the climate change discourse, and we filled the cell whose column name and row name were among the top 50 list in the climate change discourse with the frequency at which the two hashtags were associated in this discourse, and the other cells were filled with 0. This was repeated for the global warming matrix. We thus obtained two matrices with the same row and column names but different values in the cells. Then, the two matrices were input to the quadratic assignment procedure (QAP) [85] analysis provided by UCINET software [86] to assess their correlation for each year.

## 4. Results

### 4.1. General Descriptions

Association networks surrounding #climatechange and #globalwarming showed different properties. The climate change discourse included 38,821 hashtags, whereas the global warming discourse only contained 8788 hashtags. Table 1 displays the 50 most significant hashtags in the two discourses based on centrality. As some hashtags were used in the form of an abbreviation or phrase, explanations are provided in the table. Two networks shared 32 out of the 50 most significant words. Hashtags “canada”, “cdnpoli”, “sdgs”, “biodiversity”, “education”, “environmental”, “cop24”, “sustainable”, “auspol”, “food”, “agriculture”, “cleanenergy”, “renewableenergy”, “renewables”, “emissions”, “coal”, “fossilfuels”, and “cop21” only showed up on the top 50 list of the “climate change” network. Hashtags “tcot”, “california”, “p2”, “nyc”, “snow”, “agw”, “summer”, “global”, “winter”, “india”, “planet”, “heatwave”, “hoax”, “nasa”, “algore”, “world”, “oil”, and “eco” were unique on the top 50 list of the global warming network. The two lists only shared three out of the top five hashtags. In the #climatechange network, “climateaction” was ranked third place and “sustainability” was ranked fourth place, whereas they were ranked significantly lower, 17th and 22nd, respectxively, in the #globalwarming network. In the #globalwarming network, “earth” and “weather” were among the top five nodes, whereas they were ranked 14th and 24th in the #climatechange network, respectively.

### 4.2. Association Network Analysis

**Figure 2:**
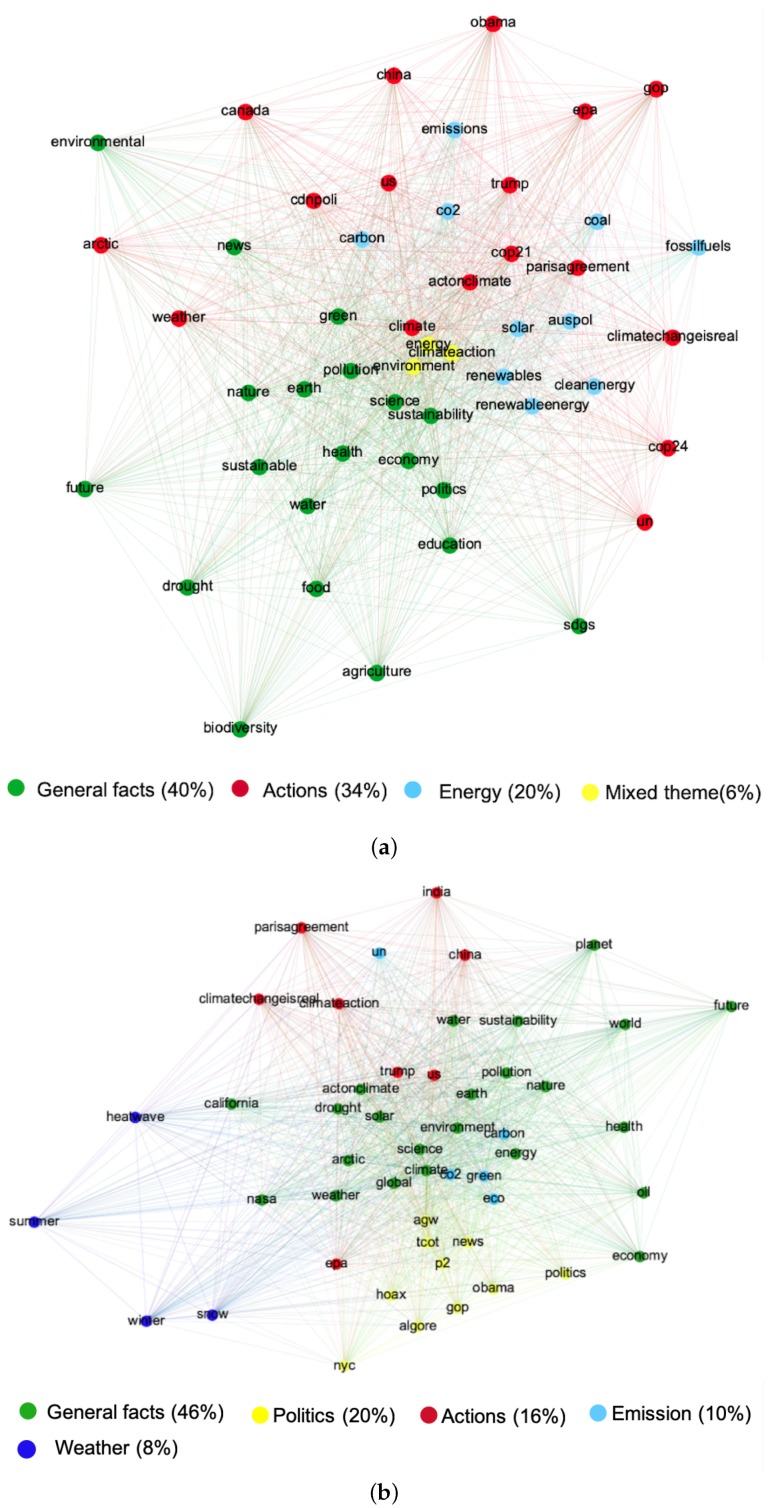
Association network of climate change discourse (**a**), and (**b**) association network of global warming discourse (**b**).

The association networks of #climatechange and #globalwarming are shown in Figure 2. Nodes are labelled with the hashtags and the undirected edges are weighted to reflect the frequency of co-occurrence. The modularity analysis identified four clusters in the #climatechange network and five in the #globalwarming network, where clusters are differentiated by color (resolution is 0.75 for climate change and 0.85 for global warming). The theme, top hashtags, and the proportion of each cluster are also summarized and represented in the network depicted in Figure 2.

The largest cluster (green nodes) of both #climatechange and #globalwarming network refer to general facts about global climate issues, sharing words about the causes or effects concerning sustainability. The difference is that the largest cluster of #globalwarming (46% of the network) includes more slogan words, such as “world”, “planet”, “global”, and “climatechangeisreal”, whereas the largest cluster of #climatechange (40% of the network) tends to discuss some specific problems, such as agriculture, biodiversity, education, and politics.

For the climate change discourse, the second-largest cluster (34%) is indicated in red and focuses on the responsibility to tackle climate change, where several global action hashtags are included, such as “un”, “parisagreement”, “cop21”, and “cop24”. The theme of the third largest cluster (20%) in the climate change discourse was energy (in blue). The smallest cluster (6%) in yellow sits in the central part of the network with a mixed theme composed of three highly ranked hashtags, including “environment” (No. 2), “climateaction” (No. 3), and “energy” (No. 6).

In the global warming network, politics was the second-largest discourse cluster (20% of the network), where “tcot”, short for “Top Conservatives on Twitter”, was the node ranked highest, and “p2”, short for “Progressives 2.0”, is also included. Several political figures, such as Obama and Al Gore, are frequently mentioned. Action toward the global climate issue was the third-largest cluster (16%), including both domestic efforts, such as “us”, “trump”, “climatechangeisreal”, “climateaction”, and “epa”, and two international items, like “china” and “india”. The fourth cluster (in blue) referred to emissions, including hashtags like “co2”, “green”, and “carbon”. The smallest cluster (8%) was composed of “snow”, “winter”, “heatwave”, and “summer”, referring to the temperature abnormalities on the earth.

### 4.3. Temporal Analysis of the Associations in the Two Discourses

The online presentations of the climate change and global warming discourses are dynamic. As shown in Table 2, for the global warming discourse, 11 key concepts remained in the top 50 central hashtags each year for all 10 years, with 16 for the climate change”discourse. By comparing the 11 nodes of the global warming discourse and the 16 nodes of the climate change discourse, we found that the two lists shared nine concepts. We found “pollution” and “earth” were unique to the keyword list of the global warming discourse, and “economy”, “water”, “china”, “coal”, “solar”, “sustainability”, and “food” only occurred on the critical list for the climate change discourse.

**Figure 3:**
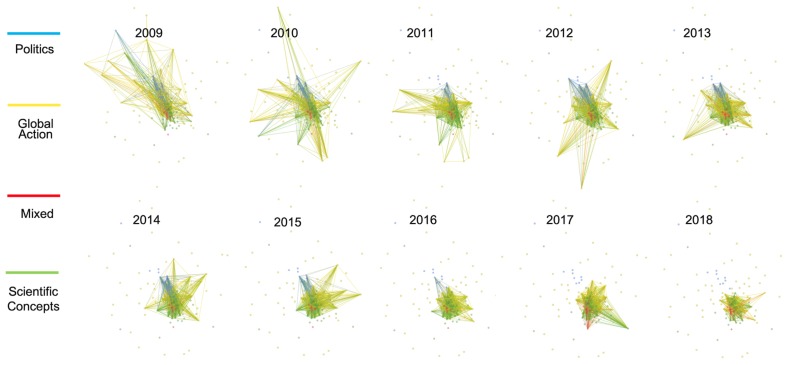
Association network of top 50 nodes of climate change for each year from 2009 to 2018.

**Figure 4:**
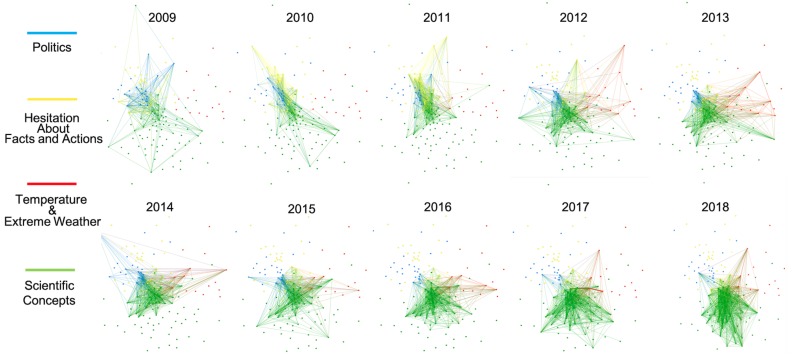
Association network of top 50 nodes of global warming for each year from 2009 to 2018.

**Figure 5:**
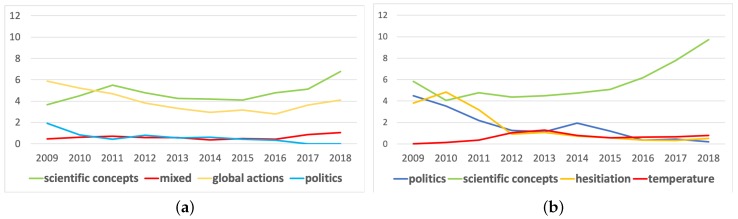
The sum of centrality for nodes in four clusters in the climate change discourse from 2009 to 2018 (**a**); (the sum of centrality for nodes in four clusters in the global warming discourse from 2009 to 2018 (**b**).

Figure 3 and Figure 4 show the overall evolution of critical hashtags’ associations in the 10-year period, where the nodes in the 10 graphs are located in the same position but the strength of associations varies across longitudinal time. Vector graphics with the label of nodes are provided in the Supplementary Materials. Four themes were identified in each discourse according to the nodes’ associations. To more explicitly demonstrate the relative importance of each cluster in each year, we calculated the sum of the degree centrality of all the nodes belonging to each cluster and their change in centrality over the 10 years, as shown in Figure 5.

Figure 3 depicts the associations of hashtags in the climate change discourse for each year from 2009 to 2018. The scientific hashtags cluster (in green) was the most important theme in the climate change discourse, especially more recently. However, some scientific hashtags, such as “ghg” (greenhouse gas), “co2”, and “forests”, were not identified in the scientific cluster but in the global actions cluster (in yellow) because these hashtags were frequently used in the global action context and identified with a closer semantic association to global action by Gephi. In addition to these hashtags, the global action cluster included a series of international activities, such as “ipcc” (Intergovernmental Panel on Climate Change), “unfccc” (United Nations Framework Convention on Climate Change), and “cop” (Conferences of the Parties) for almost every year. The blue cluster includes to political hashtags, such as “uniteblue”, “sgp”, “p2”, and “tcot”. In 2017 and 2018, the associations with political hashtags disappeared among the top 50 hashtags. The small red cluster had a mixed theme, combining “technology”, “innovation”, “education”, “africa”, “healthcare”, and “politics”. The centrality sum of the nodes in the red cluster remained rather low throughout the 10-year period but obviously increased in the last two years of the period according to Figure 5a.

Figure 4 describes the evolution of concepts’ associations in the global warming discourse during the 10 years. The red cluster included concepts such as “2012”, “hot”, “summer”, “elnino”, and “snow”, describing the weather abnormalities related to global warming. A notable finding is that before 2012, global warming’s association with temperature abnormalities and extreme weather was not salient, then the associations suddenly strengthened in 2012 when numerous hashtags about phenomena were included in the discourse. Notably, the red node in the top right-hand corner named “2012” refers to the Maya prediction that the year 2012 would be the end of the world and that the world would be destroyed by extreme natural events, and was linked to other climate hashtags for the first time in the graph exactly in 2012. The blue nodes included the political hashtags, such as “maga”, “ows”, “p2”, “tcot”, and “obama”. The involvement of political hashtags in the global warming discourse was significantly higher than that in the climate change discourse according to the comparison between Figure 5a,b. From 2009 to 2018, the number of associations with political hashtags (blue nodes) faded, as shown in Figure 4, and its importance in the semantic network gradually decreased, as shown in Figure 5, except for variation in 2014. The yellow nodes describe the hesitation about climate facts and actions, where words describing global efforts, such as “ipcc”, “cop15”, and “un”, and words questioning global warming, such as “hoax” and “fraud”, were both included. The associations between the yellow nodes were most salient in 2010 and 2011 but were less dominant in the following years. The green nodes occupied 50.7% of all the nodes representing talk about the scientific hashtags of climate issue, including words such as “ecology”, “ocean”, and “cleanenergy”. Associations between scientific hashtags (green nodes) exploded and the centrality sum of this cluster also showed an obvious rising trend in dominating the theme of the global warming discourse, according to Figure 5.

As the climate change and global warming discourses evolved over the past years, their relative statuses in public discourse also changed. Although from 2009 to 2018, increasing numbers of people started to use Twitter, resulting in an overall rise in the number of tweets and hashtags, the ratio of #climatechange frequency and #globalwarming frequency still indicated the public’s change in frame preference. Figure 1a displays that in 2009, the number of tweets with #climatechange was 2.69 times that of the tweets with #globalwarming, whereas the ratio significantly since 2013 and reached 13.02 in 2018. The climate change network showed a stronger ability to incorporate diverse hashtags into discussions, according to Figure 1b. In 2009, the hashtags that co-occurred with #climatechange were 2.44 times those that co-occurred with #globalwarming, and the ratio climbed to 6.36 in 2018.

**Figure 6:**
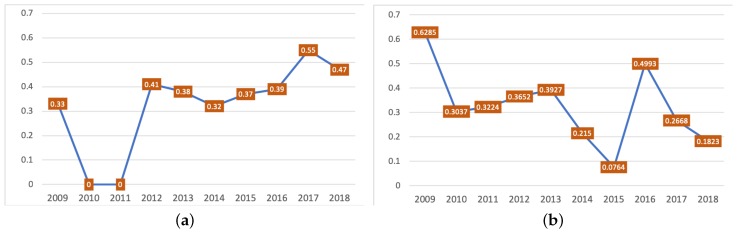
Rank order correlation between hashtags in the climate change and global warming discourses from 2009 to 2018 (**a**); correlation between matrices of the climate change discourse and the global warming discourse from 2009 to 2018 (**b**).

The rank–order correlation coefficient of nodes between the two networks maintained a stable level and showed a slight climbing trend starting 2009, as shown in Figure 6a, except for 2010 and 2011, when the *p*-values were larger than 0.05 and no significant correlations were identified. The QAP analysis showed that the associations between the two discourses were correlated in the 10-year period (the *p*-value for 2015 was 0.011; *p*-values for all the other years were less than 0.001). Figure 6b reveals that the similarity of associations between the top 50 nodes in the two discourses fluctuated and did not show a rising trend with the correlation of nodes’ rank order.

## 5. Discussion

### 5.1. Themes and Structure of the Two Discourses

#### 5.1.1. Phenomenon vs. Mechanism of Action

Climate change and global warming have long been two competing frameworks shaping the public’s perceptions, memory, and interpretations of climate issue by highlighting different aspects of issues and re-constructing them differently. By comparing the persistent words used related to the two discourses in the 10-year period in Table 2, we think that global warming showed a relative preference toward general descriptions or slogans, such as “earth” and “pollution”, whereas “climate change” was more associated to specific issues like “solar”, “coal”, “china”, and “food”.

Studies have suggested that the public shows a preference for scientific publications with general keywords compared with those with complicated scientific jargon [47], lacking a deep understanding of the complicated issue [46] and the necessity for mitigation of the climate issue [47]. These conclusions seem to suit global warming more than climate change according to the current study, which is probably because climate change receives more publicity and recognition than global warming in the scientific community. In the association network shown in Figure 2, global warming was found to be more connected with temperature abnormalities. This finding is in accordance with studies reporting that short-term temperature anomalies [87] can increase the public’s belief about global warming by increasing the understanding of this abstract issue [88], although scientists mostly make judgments based on long-term weather statistics [89]. However, none of the four words, “snow”, “summer”, “winter”, or “heatwave” in the temperature theme of global warming were ranked in the top 50 nodes list of the climate change network.

Even when climate change and global warming shared concern about similar topics such as the cause of the climate issue, global warming tended to focus on carbon emission phenomena, whereas climate change preferred a more in-depth perspective, highlighting the importance of global action to mitigate the climate issue in its second-largest cluster, with energy structure as the contributor to carbon emissions in its third largest cluster. As invisible causes and disbelief in actions have long been regarded as two key reasons for low climate concern [90], the two terminologies’ differences in connotations suggest that introducing these absent sub-topics into global warming discourse or highlighting climate change for its inherent connotations may help communicators raise public concern about climate.

#### 5.1.2. Political Connotations

Studies noted that frame preference between climate change and global warming reflects individuals’ ideological spectrum, where climate change and global warming were favored by the liberals and conservatives, respectively [10]. The cluster analysis of the semantic network in the current study demonstrated that global warming triggered far more political responses than climate change. The second largest cluster of global warming was politics-based, where hashtag “tcot”, favored by right-leaning users and “p2”, favored by left-leaning users, were both ranked in the list of top nodes of the global warming discourse, but neither was included in the list of top nodes of the climate change discourse. Considering that earlier findings suggested that global warming was more likely to be used by conservatives to question the reality of climate issue [11] and climate change is more commonly adopted when discussing action against the climate change issue [5], global warming had a stronger political connotation in public discussion.

#### 5.1.3. Discourse Structure

In the discourse surrounding #climatechange, “environment”, “energy”, and “global action” represented the themes of the three largest clusters in the network. However, three popularly recurring hashtags, “#environment”, “#energy”, and “#climateaction”, did not belong to any of the three clusters above, but formed another small tight cluster together, sitting in the most central part of the semantic network, as shown in Figure 2b. As each of the three hashtags can almost represent one sub-theme of the climate change topic and these three hashtags were tightly bundled might indicate an attempt by #climatechange users to address all three communities together [91], consolidating climate change as a topic rather than a loosely organized topic. Previous communication studies also confirmed hashtags’ function of serving as a hybrid forum [68], where heterogeneous individuals coordinate to solve problems at various levels of different domains [92]. No similar mechanism was observed in the global warming discourse, suggesting that global warming was less evocative of diverse adjacent sub-themes.

### 5.2. Evolution of Associations in the Two Discourses

#### 5.2.1. Shrinking of Traditional Political Discussions and Emergence of Discourse Alliance

The temporal analysis revealed the evolution of public discourse from 2009 to 2018. In both discourses, political discussions gradually faded, indicating a trend in the public perception of climate issues to seeming slowly return to the scientific dimension in general. In 2009, political discussion was the second most important topic in the global warming discourse, but its ranking fell to last in 2018. In the climate change discourse, as shown in Figure 3, the association to political hashtags disappeared in the recent two years. These findings seem to contradict earlier studies suggesting noticeable political polarization in the climate discussion [66,93] and a Pew report saying that the partisan divide had widened in terms of attitudes toward climate change issues [94]. We suggest a probable reason responsible for this contradiction: Although political clusters in both discourses were shrinking, in the climate change discourse, the proportion of red associations started to increase in recent years, as shown in Figure 5a. The red cluster is a topic cluster with blurred edges and is composed of diverse hashtags, such as “healthcare”, “education”, “poverty”, “innovation”, and “politics”. We examined the tweets with these co-occurrences and found that although few typical political hashtags such as “p2” and “tcot” were used, most of these tweets had strong political intentions. For example, when the public noticed that whitehouse.gov no longer provided specific pages for climate change as well as healthcare, LGBT rights, and civil rights in January 2017 [95], the red cluster expanded, as shown in Figure 5a, and a large amount of co-occurrences among these issues emerged to blame the government’s wrong practices of neglecting all these issues. We observed that “climatechange” occurred often with hashtags such as “education”, “poverty”, “economy”, “leadership”, and “innovation” as the object of the verb “rethink” in a large amount of tweets arguing for the government’s reflections on several domestic policies. Thus, we think that climate politics did not disappear but changed their form in public discussions in the last two years of the studied decade. A kind of discourse alliance formed among climate change and several other domestic political issues to show political appeal together, where a trend of pan-politicization rather than de-politicization might be identified in climate change discussion.

#### 5.2.2. Strengthened Associations between Global Warming and Weather Abnormalities

Although global warming is linked to abnormal weather phenomena, temporal analysis suggests their associations were not innate or changeless. An increase in association strength was observed in 2012 according to Figure 4. Two reasons may have contributed to this change. Firstly, the Hurricane Irene and Hurricane Sandy, two serious extreme weather events in 2011 and 2012, were proven to raise the volume of climate-related tweets and evoke the public’s consciousness of global warming [2] to some extent. However, Sandy may not be the only reason. Studies that analyzed the discourse of three extreme events on Twitter pointed out that political and ideological debates, rather than phenomenon-related discussions, dominated during Sandy, evidenced by hashtag “#sandy” not being even to the temperature and extreme weather cluster (red in Figure 4), but was identified to the cluster describing hesitation between climate facts and actions (yellow in Figure 4).

After examining every associated node in the red cluster in 2012, we suggest that the hashtag “2012” in the top right-hand corner, which is the only event-based hashtag, can provide another hint about why the associations related to temperature and extremeweather significantly increased in 2012. The Maya inscriptions about the end of the world in December 2012 were prevalent then, and even inspired a famous American disaster film named “2012”, telling a story that the earth would be destroyed by a series of disastrous extreme natural events. Previously, historians focused on the correlations between climate issues and the collapse of the Mayan civilization [96,97], but no studies have noticed that the Maya inscription about doomsday, which seemed rather ridiculous for scientists, might lead to unexpected public associations with climate issues. However, science fiction may influence the public’s attitude toward scientific issues. Frankenstein’s monster, a well-known fictional character who was a human-built creature in the novel written by Mary Shelley, has long been linked to transgenic technology by referring genetically-modified food as “Frankenstein Food” [98]. Scientists found that these associations successfully symbolized the the public’s uncertainty about the risk of transgenic technology, anxiety toward the human-made living creature, and moral discomfort about reducing life to genetic code [99], even though people all know Frankenstein was only a fictional character created 100 years ago. In the current study, we concludd that a similar mechanism may exist in global warming communication. Though “the end of world in 2012” and its adapted popular movie sounded unconvincing for scientists, the public, especially who have limited scientific literacy, were defenceless against fiction [100]. Some of the public may accept the indications of temperature rise and extreme weather, and cannot help but strengthen their associations with global warming. However, no similar associations were discovered in the climate change discourse in 2012, which may suggest that global warming is more likely to be associated with disasters, risk, or negative sentiment compared with climate change.

### 5.3. Discrepancy between the Two Discourses

The status of the two discourses varied significantly in the more recent years in the study period. Data from Google in prior study suggested that the search record for global warming was larger than that of climate change in earlier times [13]. The authors found that in the battle to be the most representative hashtag for global climate concern, #climatechange showed growing popularity and became an overwhelming trending topic compared with #globalwarming. Also, #climatechange showed a stronger ability to incorporate diverse hashtags into its discourse in both relative and absolute dimensions. Comparatively, the popularity of the global warming discourse among social media users did not increase apparently in terms of tweets volume and hashtag diversity, especially when considering the yearly increase in Twitter users. The reason for the observed shift in public discourse toward climate change from global warming may be attributed to the high exposure of climate change in the media and scientific reports in recent years [13]. Previous studies noted that perceived scientific consensus can increase acceptance of science [101]. Though global warming has been commonly used since the 1980s to describe the world-wide temperature rise, climate change is preferred by scientists to refer a range of complex changes of climate [102]. Pew found science-related accounts draw millions of followers on Facebook and volume of posts they released climbed in past years [103]. Climate scientists are found to be opinion makers on Twitter [104]. As social media has become an emerging platform for science popularization, scientific community might contribute to the prevalence of climate change discourse by talking about climate change facts and mitigating measures [75].

However, differences between two discourses were not eliminated. Even though two discourses showed more similarities in the rank order of key concepts, the QAP analysis of two matrices of semantic network showed that two discourses still embody distinct public perceptions of climate issues by associating these hashtags in different manners.

To be specific, although “ipcc”, “cop”, and “un” were mentioned in both discourses (yellow in Figure 3 and Figure 4) in earlier years, the clusters to which they belonged had significantly different meanings. As mentioned in the results section, these hashtags were associated with a series of scientific hashtags in the climate change discourse, appealing to global efforts. In the global warming discourse, they were clustered with “hoax” and “frame”, showing lack of belief in climate issue facts and hesitation about global efforts. More recently, when discussions about temperature, politics, and hesitation significantly shrank in the global warming discourse, the wo discourses showed more similarities about the importance of scientific concepts according to Figure 5a,b. However, links between global efforts and scientific facts were not constructed in the global warming discourse. According to a network model for cognition, the lack of associations means fewer psychological activations will spread to make global action salient for people talking about global warming than people talking about climate change [40], even though the facts of climate issues are highly recognized in both discourses.

## 6. Conclusions

As social media is gradually overtaking the role of legacy media providing a forum for public discussion, the semantic associations contained in social media discussions reflect and reinforce how individuals portray global climate issues. By examining hashtag co-occurrence patterns on Twitter between 2009 and 2018, we identified distinct climate perceptions hidden behind two competing climate discourses and discovered how these two discourses evolved.

We found that broad scientific, social, political, and international discussions are the topics of public climate discourse. Although the semantic difference between climate change and global warming seems subtle, the differences in their cognitive associations are not trivial. Despite some shared concerns between the two discourses, “global warming” is more politicized and focuses more on general phenomena, especially temperature abnormalities, whereas climate change is a more compact topic with a more scientific perspective and tends to refer to specific issues. The temporal analysis revealed that traditional political discussions decreased in both discourses but climate change started to build a discourse alliance with diverse domestic issues to show political intentions. Global warming’s associations to extreme events and temperature change were suddenly strengthened around 2012. Climate change is becoming dominant compared with global warming in public discussions. Although the two discourses are becoming increasingly similar in the rank order of climate concepts, a notable discrepancy still exists in the way in which they get concepts associated. These observations may provide climate communicators with theoretical and practical hints to narrow the discrepancy between diverse climate perceptions.

### Limitation and Future Directions

Though big data allowed us to decrease the bias by dealing with the whole set of social media data rather than samples, discrepancies still exist between social media users and the public. As most Twitter users do not disclose their age, education, income, and gender in users’ profile, demographics were not introduced as moderator factors in this study. Previous studies noted that in 1970s, global cooling was a prominent climate concern amongst the public [105]. While in the 1980s, ozone layer depletion, species extinction and rainforest destruction became salient on the mass media agenda [106]. Considering the historical background of climate issues, age might influence how individuals perceive climate issues. According to the statistics in 2017 [107], only 16 % of older people (older than 60) in America use Twitter, while the proportion is 39% for people between 30–59 years old and 47% for people younger than 30 years old (Stastista, 2017). Our results reflect the climate perception of older people who use Twitter, as well as younger people amongst whom Twitter is more popular. Although some scholars reported that it is statistically reliable to take data on Twitter as a substitute and supplement for polling [108], we thought our results should be further examined before being generalized to the whole population.

In this study, we characterized the differences between two popular climate discourses and examined how two discourses evolved over a 10-year period. We did not focus on the interactions between public climate discourse and external factors. However, the evolution of climate discourse might be driven by several external forces such as scientific efforts, natural events, politics and online information (or misinformation) campaigns. The prevalence of certain climate concepts may inverse be weaponized to cause rhetorical shifts in politics and science popularization. For instance, previous studies noted that in the 2016 U.S. Presidential Election, state-supported misinformation campaigns took place to manipulate public opinion [109] and fake accounts were involved in spreading low-credibility news on Twitter [110]. How social media climate discourse reflects and interacts with other sub-systems of our society should be noticed and explored in future. More studies like [2], who examined the influence of several extreme events on public climate change perception, should be conducted to reveal the interactions between public discourse and natural, scientific, social, or political events. In particular, factors promoting public consensus and factors resulting in discourse discrepancy should be further identified to help climate communicators narrow the public cognitive divergence about the global climate issue. 

## Figures and Tables

**Table 1:** The top 50 central hashtags on Twitter surrounding #climatechange and #globalwarming from 2009 to 2018. The hashtag with * is explained in Appendix A in ascending alphabetical order.

No.	#Climatechange		#Globalwarming	
	Hashtag	Centrality	Hashtag	Centrality
1	climate	0.466	climate	0.530
2	environment	0.465	environment	0.446
3	climateaction	0.391	science	0.319
4	sustainability	0.316	earth	0.296
5	science	0.314	weather	0.280
6	energy	0.283	us *	0.280
7	trump	0.257	trump	0.263
8	us *	0.247	pollution	0.256
9	cop21 *	0.232	co2	0.244
10	parisagreement *	0.232	green	0.239
11	actonclimate *	0.225	tcot *	0.229
12	water	0.221	nature	0.213
13	pollution	0.210	news	0.198
14	earth	0.207	energy	0.192
15	green	0.200	climatechangeisreal	0.187
16	climatechangeisreal	0.195	obama	0.181
17	renewableenergy *	0.194	climateaction	0.175
18	health	0.193	algore *	0.174
19	nature	0.187	water	0.171
20	renewables	0.186	agw *	0.164
21	cleanenergy	0.176	carbon	0.164
22	carbon	0.175	sustainability	0.163
23	co2	0.174	snow	0.161
24	weather	0.169	world	0.157
25	solar	0.165	gop *	0.156
26	economy	0.164	arctic	0.150
27	auspol	0.163 *	winter	0.145
28	education	0.155	p2 *	0.144
29	news	0.152	drought	0.142
30	drought	0.150	epa *	0.141
31	coal	0.147	global	0.137
32	sustainable	0.147	eco	0.137
33	cdnpoli	0.144 *	actonclimate	0.136
34	sdgs	0.143 *	health	0.134
35	china	0.143	un *	0.133
36	gop	0.143 *	solar	0.132
37	food	0.141	economy	0.131
38	un	0.141 *	hoax	0.131
39	cop24 *	0.140	california	0.130
40	agriculture	0.138	politics	0.129
41	environmental	0.136	india	0.128
42	fossilfuels	0.134	china	0.127
43	arctic	0.134	planet	0.127
44	epa *	0.133	parisagreement *	0.126
45	biodiversity	0.132	heatwave	0.125
46	future	0.131	summer	0.121
47	canada	0.128	nyc *	0.118
48	emissions	0.128	nasa	0.118
49	obama	0.127	future	0.118
50	politics	0.125	oil	0.117

**Table 2:** Hashtags that remained on the top 50 list for the climate change or the global warming discourse from 2009 to 2018.

	Unique	Shared
#climatechange	china, solar, water, food, economy, coal, sustainability	co2, news, carbon, green, climate,
#globalwarming	pollution, earth	us, energy, science, environment

## Supplementary Materials

The following are available online at https://www.mdpi.com/1660-4601/17/3/1062/s1.

## Appendix A

#agw, short for anthropogenic global warming, indicating global warming is caused by human activities.

#cdnpoli, short for Canadian politics

#cop21, the yearly session of COP (short for the Conference of the Parties) held in 2015.

#cop24, the yearly session of COP (short for the Conference of the Parties) held in 2018.

#epa, short for the United States Environmental Protection Agency founded in 1970, an agency aiming at protecting environment.

#gop, short for Grand Old Party, the Republican political party in the United States.

#nyc, short for New York City

#p2, short for Progressives 2.0, a hashtag used to show progressive political standpoints on Twitter.

#parisagreement, Paris Agreement, the agreement signed on UNFCCC in 2016 to deal with global warming by reducing greenhouse gas emissions.

#sdgs, short for Sustainable Development Goals, containing 17 global goals put forward by the United Nations General Assembly in 2015 and expected to be achieved in 2030.

#tcot, short for Top Conservatives On Twitter, a hashtag used to show conservative political standpoints on Twitter.

#un, short for the United Nations

#us, short for the United States
